# Social bonds provide multiple pathways to reproductive success in wild male chimpanzees

**DOI:** 10.1016/j.isci.2021.102864

**Published:** 2021-08-17

**Authors:** Joseph T. Feldblum, Christopher Krupenye, Joel Bray, Anne E. Pusey, Ian C. Gilby

**Affiliations:** 1 Department of Anthropology, University of Michigan, Ann Arbor, MI 48109, USA; 2 Society of Fellows, University of Michigan, Ann Arbor, MI 48109, USA; 3 Department of Evolutionary Anthropology, Duke University, Durham, NC 27708, USA; 4 Department of Psychological & Brain Sciences, Johns Hopkins University, Baltimore, MD 21218, USA; 5 Department of Psychology, Durham University, Durham, UK; 6 School of Human Evolution and Social Change, and Institute of Human Origins, Arizona State University, Tempe, AZ 85287-2402, USA

**Keywords:** Ecology, Biological sciences, Zoology, Ethology

## Abstract

In most male mammals, fitness is strongly shaped by competitive access to mates, a non-shareable resource. How, then, did selection favor the evolution of cooperative social bonds? We used behavioral and genetic data on wild chimpanzees (*Pan troglodytes schweinfurthii*) in Gombe National Park, Tanzania, to study the mechanisms by which male-male social bonds increase reproductive success. Social bonds increased fitness in several ways: first, subordinate males that formed strong bonds with the alpha male had higher siring success. Independently, males with larger networks of strong bonds had higher siring success. In the short term, bonds predicted coalition formation and centrality in the coalition network, suggesting that males benefit from being potential allies to numerous male rivals. In the long term, male ties influenced fitness via improved dominance rank for males that attain alpha status. Together, these results suggest that male bonds evolved in chimpanzees by affording both short- and long-term pathways to reproductive success.

## Highlights

• Three independent pathways linked male chimpanzee sociality and siring success
• Alphas, but not others with high rank, sired a disproportionate share of offspring
• Subordinates with strong bonds with the alpha had better siring success
• Subordinates with many strong ties to other subordinates had better siring success

## Graphical abstract

**Figure:**
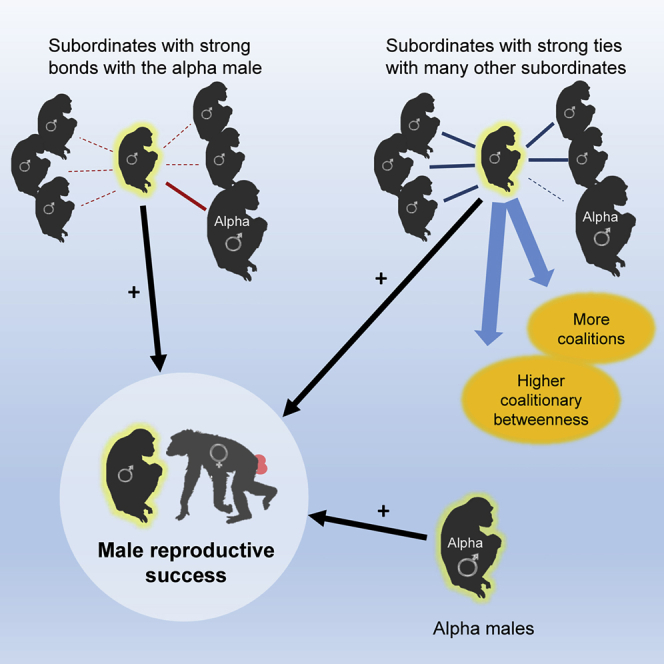

## Introduction

Humans and other animals form differentiated affiliative bonds that are associated with survival and reproduction (Holt-Lunstad et al., 2010; Ostner and Schülke, 2018; Seyfarth and Cheney, 2012). Yet despite growing evidence of fitness consequences of sociality, only two studies in non-human mammal species have provided evidence of the mechanisms by which sociality might lead to higher survival or reproductive success (Ostner and Schülke, 2018; Schülke et al., 2010; Thompson, 2019). In wild horses (*Equus caballus*), more social mares experienced less frequent male harassment and had higher rates of offspring production (Cameron et al., 2009). In Assamese macaques (*Macaca assamensis*), males that had stronger bonds formed more aggressive coalitions, rose in rank in subsequent periods, and ultimately sired more offspring (Ostner and Schülke, 2018; Schülke et al., 2010). Here, we use long-term behavioral and genetic data to investigate the relationship between sociality and reproduction in male chimpanzees.

Previous studies in male chimpanzees provide reason to suspect similar mechanisms at work. Subordinate males that support and affiliate with the alpha male have higher mating success (Bray et al., 2016; Duffy et al., 2007). Additionally, male affiliative behavior can predict coalitionary support in aggressive interactions (Mitani, 2006; Watts, 2002), and subordinate males with higher direct and indirect centrality in the aggressive coalitions network have higher current and future rank (Gilby et al., 2013; Watts, 2018) and are more likely to sire offspring in the short term, independent of dominance rank (Gilby et al., 2013). Finally, males with more strong affiliative ties are more likely to rise in rank (Bray et al., in press), and higher-ranking males sire more offspring (Boesch et al., 2006; Langergraber et al., 2013; Newton-Fisher et al., 2010; Wroblewski et al., 2009). Yet no study in chimpanzees has demonstrated a link between social relationships and reproductive success. Our study thus aims to fill gaps in this picture of male cooperative and competitive behavior by determining (1) whether male social bonds predict reproductive success and (2) what mechanisms link social bonds to coalition formation and paternity. Specifically, we tested three hypotheses regarding the mechanisms linking sociality and paternity success. These were that (1) subordinate males can form strong bonds with the alpha male to achieve greater reproductive success via mating concessions, which we call the “alpha concessions” hypothesis; (2) males can form bonds with other males to improve their short-term reproductive success via social leverage stemming from aggressive coalitionary support, which we call the “coalitionary support” hypothesis; and (3) males can form bonds with other males to increase their long-term reproductive success via improved dominance rank, which we call the “rank improvement” hypothesis.

## Results

Using model comparisons, we first tested whether male sociality was associated with an increased likelihood of siring offspring. From a dataset of 56 siring events with known paternity between 1980 and 2014, we constructed a null model with a binary outcome variable indicating siring success and terms for male age, male genetic relatedness to the infant's mother, male dominance rank, and random intercepts for male identity (see STAR Methods). We then added measures of association and grooming to determine if they improved model fit. Because studies differ over the most biologically meaningful method for characterizing social connectedness (Ellis et al., 2019; Ostner and Schülke, 2018; Silk et al., 2013; Thompson, 2019), we conducted information-theoretic comparisons of models containing all terms from the null model plus measures based on (a) dyadic association in small groups (hereafter “association”), (b) grooming relationships and overall grooming effort, as well as (c) composite indices of dyadic association and grooming (see STAR Methods and Table 1 for a full list and descriptions of model terms). We found that two models had better fit than the null model: one containing the number of “strong association ties”, defined as dyadic association indices above the yearly community mean (Ellis et al., 2019), and a second containing the summed strength of strong dyadic association ties (ΔAICc = −7.35 and −4.63, respectively; Table 1). These two measures were highly correlated (Pearson correlation = 0.89) and likely represent two ways of capturing the same social dynamic. According to the best model, males with more strong association ties had a higher likelihood of siring offspring (binomial generalized linear mixed model, odds ratio [OR] = 1.63, 95% confidence interval [CI] = [1.19–2.23]); remarkably, a one standard deviation increase in the number of strong association ties correspondeds to a 63% increase in probability of siring a given offspring, after accounting for male age, relatedness to the mother, and dominance rank score (see STAR Methods; Figure S1). Even after adding an additional term to control for overall gregariousness (the total time each male spent with other males and females of reproductive age; see STAR Methods), the same two models had better fit than the null model (ΔAICc = −3.75 and −1.51, respectively), and in the best-fit model, the count of strong association ties was again positively associated with the likelihood of siring offspring for males (OR = 1.52, CI = [1.08–2.15]). Results also held when using raw association counts instead of sociality indices based on association (see STAR Methods, Table S1).

**Table 1:** List of models and model fit parameters for full dataset, using measures of social relationships to predict male siring success

Affiliative index	Model	Df	AICc	ΔAICc	Akaike weight
DAI4	Count of strong association ties	6	360.104	−7.349	0.700
DAI4	Sum of strong assn. indices	6	362.823	−4.63	0.180
(none)	Null model	5	367.453	0	0.018
DAI4	Count of all association ties	6	368.384	0.931	0.011
DAI4	Sum of all association indices	6	369.129	1.676	0.008
Grooming	Total grooming partners	6	368.668	1.215	0.01
Grooming	Total time spent grooming	6	368.679	1.226	0.01
Grooming	Sum of grooming times above mean	6	368.777	1.324	0.009
Grooming	Count of strong grooming rate partners	6	369.085	1.631	0.008
Grooming	Sum of grooming rates above the mean	6	369.288	1.835	0.007
Grooming	Overall grooming rate	6	369.388	1.935	0.007
Grooming	Count of strong grooming time partners	6	369.468	2.015	0.006
Grooming	Mean grooming rate across all partners	6	369.485	2.032	0.006
CSI	Sum of top 3 CSI values	6	369.282	1.828	0.007
CSI	Sum of CSI values above the mean	6	369.465	2.011	0.006
CSI	Count of high CSI ties	6	369.471	2.018	0.006

Models include all terms from the null model, plus the term described in the model column. ΔAICc shows difference in corrected AIC score between each model and the null model, with negative values indicating models that fit better than the null model and positive values indicating those that fit less well. The two best fit models account for 88% of total model weight.

**Figure 1:**
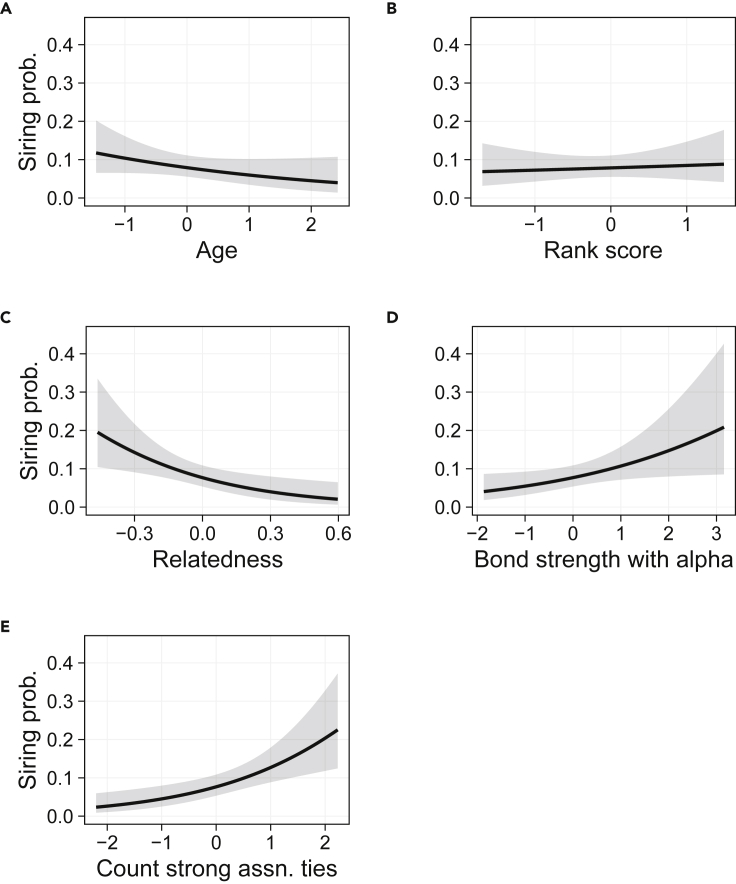
Predictors of siring success among subordinate males Predicted relationship between siring probability and (A) male age; (B) male rank score; (C) male-female genetic relatedness; (D) strength of the composite sociality index with the alpha male; and (E) count of strong association ties, among subordinate (i.e., non-alpha) males, using a restricted dataset of 45 siring events by subordinate males. Predictor variables are standardized within each period except for age, which was standardized across the entire dataset, and the genetic relatedness term. Error regions indicate 95% confidence intervals.

**Figure 2:**
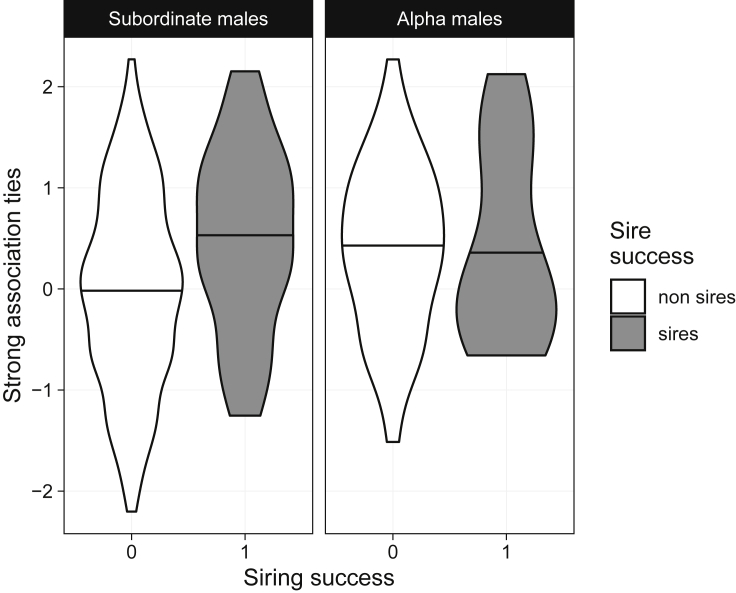
Strong association tie count and siring success Empirical distribution of scaled strong association tie count by siring success. Successful sires are shaded dark gray, while males that failed to sire offspring in a given window are shaded white. Horizontal lines within violins indicate 50% quantiles. The left-hand panel shows distributions among males ranked second or lower, while the right-hand panel shows distributions among alpha males only.

Next, we investigated possible mechanisms by which social bonds may confer fitness benefits. We first tested the alpha concession hypothesis; previous research in chimpanzees showed that subordinate males that formed strong relationships with the alpha male received mating concessions by the alpha (Bray et al., 2016; Duffy et al., 2007) (see also e.g. Henzi et al., 2010; Snyder-Mackler et al., 2012). Thus, to determine whether those subordinate males that had a strong social bond with the alpha male had higher reproductive success, we analyzed the subset of 45 siring events by non-alpha males. We again generated a null model with male age, male genetic relatedness with the mother, and male dominance rank score, with random intercepts for male identity. We then compared the null model with the model containing the number of strong association ties (the term from the best fit model in the previous analysis), models including several measures of bond strength with the alpha male, and models including both (Table 2). The best-fit model (ΔAICc = −11.70) included the composite sociality index (CSI) of grooming and association with the alpha male, as well as the count of strong association ties, while the next best fitting model included grooming rate with the alpha male, as well as the count of strong association ties (ΔAICc = −9.86; Table 2). In the best-fit model, both CSI with the alpha male (OR = 1.44, CI = 1.03–2.02) and count of strong association ties (OR = 1.76, CI = 1.24–2.48) were positively associated with likelihood of siring offspring (Figures 1, 2, and S2). Thus, subordinate males with strong bonds with the alpha male, as well as those with many strong association ties, were more likely to sire a given offspring, after accounting for the effects of dominance rank score, age, and genetic relatedness with the mother. CSI with the alpha male and the number of strong association ties were not highly correlated (Pearson correlation = 0.08; Figure S2), suggesting that they represented independent tactics for achieving siring success.

**Table 2:** List of models and model fit parameters using measures of social relationships to predict male siring success among subordinate males only

Model	df	AICc	ΔAICc	Akaike weight
CSI with alpha + count of strong association ties	7	282.489	−11.700	0.532
Grooming rate with alpha + count of strong association ties	7	284.330	−9.859	0.212
Count of strong association ties	6	284.773	−9.416	0.17
Association rate with alpha + count of strong association ties	7	286.476	−7.713	0.072
CSI with alpha	6	291.437	−2.752	0.006
Association rate with alpha	6	292.645	−1.544	0.003
Grooming rate with alpha	6	292.848	−1.341	0.003
**Null model**	5	294.189	0	0.002

Models include all terms from the null model, plus the term(s) described in the model column. ΔAICc shows difference in corrected AIC score between each model and the null model, with negative values indicating models that fit better than the null model.

To further ensure that the two measures were structurally independent, we conducted two additional analyses. First we re-calculated the number of strong association ties, this time excluding alpha males from the count. Model fits and parameter estimates were effectively identical to those presented here (Table S2). Second, we tested whether bonds with the second-ranked (beta) male had comparable effects on siring success to those with the alpha male. In contrast with alpha males, we found no evidence that strong bonds with beta males improved siring success, suggesting that bonds with the alpha male are functionally different from other close bonds (Table S3). These findings provide support for the “alpha concessions” hypothesis: male chimpanzees can gain fitness benefits by establishing strong bonds with alpha males, who are more likely to concede matings to close social partners (Bray et al., 2016). They also point to another independent strategy: males can also succeed by building a large network of strong association ties, which do not necessarily include the alpha male.

A large bond network may afford multiple additional routes to fitness by conferring social leverage that extends beyond an individual's own resource holding potential (Lewis, 2002; Watts, 2010). In the short term, social bonds may facilitate coalition formation, which could help males avoid harassment or gain access to resources such as mating opportunities (Bissonnette et al., 2015; Ostner and Schülke, 2018; Schülke et al., 2010; Thompson, 2019; Watts, 1998). Coalitions occur when two or more individuals jointly direct aggression at one or more targets (Bissonnette et al., 2015; Harcourt and de Waal, 1992) and have been shown to confer fitness advantages. For example, in Assamese macaques, social bonds predicted coalition formation, and both bond strength and coalition formation predicted rank increase and reproductive success in later periods (Schülke et al., 2010). To test the coalitionary support hypothesis in chimpanzees, we investigated whether dyads that had stronger association relationships (based, as above, on association in small parties) and/or those with stronger grooming relationships were more likely to form aggressive coalitions, accounting for dominance rank, age, time observed together (because dyads that were rarely seen together have fewer opportunities to form coalitions), and individual tendencies to form coalitions (see STAR Methods). As predicted, we found that relationship strength based on association was indeed positively correlated with the probability of forming coalitions (probit AME model, posterior mean = 0.17, 95% credible interval = [0.02–0.32]), an effect that was similar to that of grooming rate on coalition formation (posterior mean = 0.15, CI = [-0.01–0.32]) (Figure S3).

Previous research in Gombe found that it was specifically indirect ties in the network of coalition formation that predicted rank improvement and reproductive success. Males with high betweenness in the coalition network—those that formed coalitions with males that did not form coalitions with each other—were more likely to rise in rank and sire offspring (Gilby et al., 2013). Similarly, subordinate males in two smaller chimpanzee communities at other study sites achieved disproportionate mating success during periods when higher-ranking males depended on their support to consolidate power in a dominance struggle (de Waal, 1982; Nishida, 1983). We therefore examined whether males with more strong association ties also had higher betweenness in the network of male coalition formation and found this to be the case (*F* = 16.15, estimate = 0.03, 95% confidence interval = [0.01–0.04]; Figure S4). Together, these findings suggest that one explanation for the relationship between strong association tie count and reproductive success is that males with many strong association ties leverage their position as a potential ally of numerous rivals within their community to gain access to mating opportunities or protection from harassment, consistent with the coalitionary support hypothesis.

Finally, we examined the rank improvement hypothesis: that sociality facilitates long-term improvements in dominance rank, which provide future reproductive advantage (Ostner and Schülke, 2018; Schülke et al., 2010; Thompson, 2019). Bray et al. (in press) found that at Gombe, males with more strong association ties achieved higher dominance rank in the following year. Therefore, a positive association between rank and reproductive success would suggest that social bonds have a delayed effect on reproductive success via rank acquisition (Ostner and Schülke, 2018; Thompson, 2019). We found partial support for this hypothesis: using all siring events, dominance rank was positively associated with likelihood of siring offspring (best model: OR = 1.53, CI = 1.12–2.10; Figure S1). In addition, a path analysis revealed that higher rank score among subordinate males may indirectly increase their reproductive success because higher-ranking subordinates tended to have stronger bonds with the alpha (Table S4, Figure S7). However, after excluding alpha males, dominance rank was not directly associated with the likelihood of siring offspring in the best-fit model (best model: OR = 1.09, CI = 0.70–1.70; Figure 1). In addition, while alpha males sired the highest proportion of offspring during the study period (20%), males occupying each rank position from two (beta) to 10 sired roughly 7–10% of total offspring (Figure S5). Therefore, it appears that rank acquisition is a long-term reproductive strategy that primarily benefits those males that attain alpha status.

## Discussion

Among group-living animals, social bonds are expected to evolve to mitigate the inevitable costs of living in groups (Ostner and Schülke, 2018). Male chimpanzees compete aggressively for access to females (Duffy et al., 2007; Muller and Mitani, 2005; Wrangham, 2002) and alpha males tend to display the highest rates of aggression (Muller, 2002) and usually sire a disproportionate share of offspring (Boesch et al., 2006; Langergraber et al., 2013; Newton-Fisher et al., 2010; Wroblewski et al., 2009). Our results suggest that bonds formed by subordinate male chimpanzees provide leverage with which to achieve higher reproductive success in this competitive environment. Subordinate males that formed strong social relationships with the alpha male, as well as those that had many strong association ties with non-alpha males, were more likely to sire offspring than other subordinate males.

Leverage refers to the components of power relationships independent of individual resource holding potential or fighting ability, such as the fighting ability of coalition partners or the possession of a resource (like agonistic support) that cannot be appropriated by force (Lewis, 2002; Watts, 2010). Individuals should therefore gain leverage from alliances with high-ranking group mates, as well as from a position as a potential ally to two rivals. Previous research found that subordinate males gain leverage from prosocial relationships with alpha males, which in turn confers improved access to mates. Alpha males grant mating concessions to subordinate males that provide them with coalitionary support (Duffy et al., 2007) and those that groom them at high rates (Bray et al., 2016). Reproductive concessions have also been reported in other mammalian species (Henzi et al., 2010; Snyder-Mackler et al., 2012), although it is often difficult to separate the effects of reproductive concessions from those of limited control by dominant individuals (Bray et al., 2016; Clutton-Brock, 1998). Using genetic data, we have extended previous behavioral evidence in chimpanzees by showing that the strength of subordinate males' relationship with the alpha male was positively associated with their likelihood of siring offspring.

We also found that subordinate males with larger networks of strong association ties with other subordinate males were more likely to sire offspring (Figures 1, 2, and S1). The effect of the number of strong association ties was independent of the effect of the amount of time males spent with other group members in general. This supports the idea that, in species with high fission-fusion social dynamics (Aureli et al., 2008), association preferences are important measures of social bonds (Bray et al., in press; Bray and Gilby, 2020; Gilby and Wrangham, 2008). Indeed, dyads with stronger association ties were more likely to form coalitions, indicating that leverage derived from close relationships likely provides a short-term strategy linking social bonds with reproductive success via coalition formation.

Moreover, males with more strong association ties had higher coalitionary betweenness, i.e., they tended to form coalitions with males that did not form coalitions with each other. Previous research in the Gombe population found that males with high coalitionary betweenness were more likely to rise in rank and father offspring (Gilby et al., 2013). While it is possible that social temperaments and skills that influence indirect centrality in a social network could be heritable in a species with complex social cognition (Brent, 2015), it remains unlikely that coalitionary betweenness would be a target of selection because this measure also depends on the coalitionary behavior of other individuals. Our results are consistent with the more parsimonious explanation that individual behavior operating at the local network level (maximizing one's set of potential coalition partners by forming more strong social bonds) is responsible for the previously reported association between betweenness in the network of coalitions and male reproductive success. Indeed, cognitive studies report that chimpanzees are capable of understanding the concept of social leverage (Sánchez-Amaro et al., 2018) and thus that they may be able to take advantage of leverage afforded by a broad set of potential coalition partners. Our results are also consistent with earlier research in small groups of captive and wild chimpanzees, which found that males that were potential allies of two higher-ranked rivals temporarily leveraged their position for greater mating success (de Waal, 1982; Nishida, 1983). Similar results were also reported in humans in organizational settings: people that connect others that do not themselves connect have higher compensation, better performance evaluations, and gain more promotions than their structurally redundant peers (Burt, 2004, 2009), an effect attributed in part to leverage gained by the ability to form alliances with two individuals in conflict (Burt, 2009).

Finally, another study from the Gombe population found that males with more strong association ties tended to rise in rank or maintain high rank (Bray et al., in press). Here, we confirmed earlier evidence that dominance rank predicts reproductive success among male chimpanzees (Boesch et al., 2006; Langergraber et al., 2013; Newton-Fisher et al., 2010; Wroblewski et al., 2009). While higher-ranking subordinate males tended to form stronger bonds with the alpha male, when alpha males were excluded from the analysis, male dominance rank score was not an important direct predictor of siring success. In the long term, of the 36 males in the current study community that survived at least until age 16, 12 (33%) achieved alpha status at some point. Therefore, social bonds among male chimpanzees may contribute to future reproductive success by facilitating higher dominance rank if males rise to, and retain, alpha status. In the short term, however, it appears that subordinate males benefit from strong relationships with the alpha male but not directly from competitive advantages gained from higher dominance rank.

These results based on genetic data differ from earlier evidence in Gombe, which found that higher dominance rank predicted mating success even among non-alpha males (Bray et al., 2016). Additional data are needed to clarify whether these differing results are due to the smaller sample size of the current genetic dataset or to the timing of mating by subordinate males relative to the period of maximum female fecundity.

It is unclear why males that had large grooming networks or those that spent more time grooming overall did not sire more offspring in the current analysis. One hypothesis that remains to be tested is that grooming that occurs in small parties serves to establish social bonds but is recorded less frequently by observers (because focals of small groups are rare), while grooming observed in larger groups is less indicative of partner preference. This would predict that grooming data recorded in small parties would show a similar relationship with siring success as do strong association ties.

Kinship has long been recognized as a pivotal factor in the evolution of male social bonds (van Hooff and van Schaik, 1994), but more recent evidence underscores the importance of bonds among unrelated males as well (Ostner and Schülke, 2018; Schülke et al., 2010). Although male philopatry and female dispersal mean male chimpanzees tend to reside with kin, males also form bonds, cooperate, and affiliate with non-kin (Bray and Gilby, 2020; Goldberg and Wrangham, 1997; Langergraber et al., 2007; Mitani, 2009; Mitani et al., 2000). Our results may help explain this phenomenon: if males benefit from forming strong association relationships and coalitions with many males (Gilby et al., 2013), there is a clear selective advantage to building large bond and alliance networks that extend beyond close kin.

Among mammals, sociality predicts survival and longevity in males and females across numerous taxa, including cetaceans (Ellis et al., 2017; Stanton and Mann, 2012), non-human primates (Archie et al., 2014; Brent et al., 2013; Campos et al., 2020; Silk et al, 2003, 2009, 2010), hyraxes (Barocas et al., 2011), ungulates (Nuñez et al., 2015; Vander Wal et al., 2015), and humans (Holt-Lunstad et al., 2010; House et al., 1988). Several studies have also linked sociality to reproductive output, including in primates (Assamese macaques: Ostner and Schülke, 2018; Schülke et al., 2010; rhesus macaques, *Macaca mulatta*: Brent et al., 2013), cetaceans (Wiszniewski et al., 2012), and ungulates (Cameron et al., 2009). Yet other research has complicated this picture; for example, one study found that male Guinea baboons (*Papio papio*) that socialized more with other males had lower reproductive success, likely due to a trade-off between time spent socializing with males and time spent socializing with females (Dal Pesco, 2020). Notably, male guinea baboons have relatively egalitarian dominance relationships, with low rates of within-sex aggression and indistinct dominance relationships (Patzelt et al., 2014). Our results thus add to a growing body of evidence that social bonds are linked to reproductive output. Further, because male chimpanzees compete aggressively for access to females (Duffy et al., 2007; Muller and Mitani, 2005; Wrangham, 2002), our results suggest that same-sex affiliative social relationships should contribute to reproductive success in taxa with strong within-sex competition.

Few studies in mammals have investigated the mechanisms by which sociality produces improved reproductive output. In wild horses, more social mares experienced less frequent male harassment and had higher rates of offspring production (Cameron et al., 2009), and in Assamese macaques, unrelated males that had stronger bonds with their top three partners formed more coalitions, rose in rank in subsequent periods, and ultimately sired more offspring (Schülke et al., 2010). Recent reviews have called for greater attention to the mechanisms underpinning the sociality-fitness link in mammals (Ostner and Schülke, 2018; Silk et al., 2013; Thompson, 2019). Here, we provide evidence consistent with multiple independent pathways linking male social bonds and reproductive success in a social mammal.

**Figure 3:**
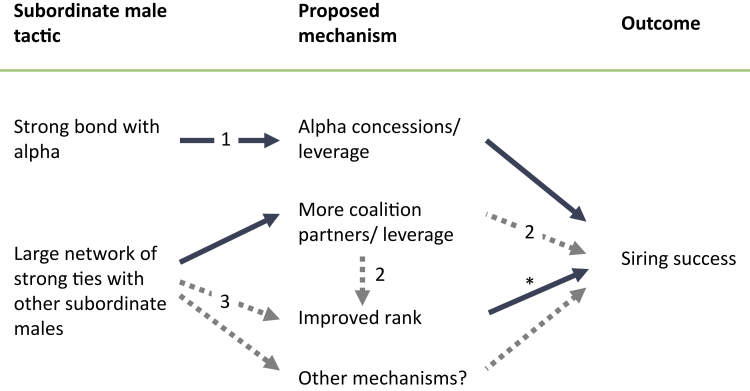
Diagram of proposed mechanisms Proposed mechanistic links between male tactics and reproductive success. Solid arrows indicate links supported by the current analysis, and numbers on arrows indicate links established in other research in Gombe. (1) Bray et al. (2016) reported that strong bonds with alpha males predicted mating success; (2) Gilby et al. (2013) reported that betweenness centrality in the coalition network predicted rank increase and siring success; (3) Bray et al. (in press) reported that males with more strong association ties showed later increases in rank score. Asterisk (∗) indicates that the current study found evidence that rank improved siring success only for alpha males, although other studies in Gombe and other sites have documented higher reproductive success for high-ranking males beyond the alpha position (see main text).

Our goal was to determine how social bonds are related to coalition formation and reproductive output. We found that male chimpanzees benefit from social bonds in three ways: first, we show that males that formed strong social bonds with the alpha male were more likely to sire offspring, probably via the previously demonstrated mechanism of mating concessions by the alpha male (Bray et al., 2016). Second, we show that males with large networks of strong ties were more likely to sire offspring. In the short term, males that formed strong association ties with many group mates had more coalition partners were more central in the network of coalition formation and were more likely to sire offspring, independent of dominance rank. In the long term, as previously shown, male chimpanzees with more strong association ties had higher rank scores a year later (Bray et al., in press), and current results demonstrate that those that rose to the alpha position had better siring success (Figure 3). Because males with more strong association ties formed more coalitions, this long-term mechanism resembles the mechanism described in male Assamese macaques (Schülke et al., 2010). Identifying the precise short-term mechanism by which strong association ties might facilitate leverage and improved male reproductive success (e.g. via coalitionary mate guarding (Watts, 1998) reduced harassment outside of mating contexts (Cameron et al., 2009), or some other mechanism) requires further research. Nevertheless, current results suggest that social bonds provide multiple short- and long-term routes to reproductive success. In doing so, they underscore the behavioral flexibility of non-human primates and broaden the scope of proximate mechanisms underlying social evolution.

### Limitations of the study

This study relied on observational data and is unable to confirm a causal relationship between male sociality and reproductive success. It remains possible that some other factor, such as phenotypic quality, determines both male social behavior and male siring success and that therefore the relationships reported in the current study are spurious. However, evidence of multiple independent pathways linking male bond formation and reproductive success make this a less parsimonious explanation.

## STAR★Methods

### Key resources table

**Table:** 

REAGENT or RESOURCE	SOURCE	IDENTIFIER
**Deposited data**		

Cleaned data and code	https://github.com/jtfeld/male_sociality_RS	Zenodo: https://doi.org/10.5281/zenodo.5027438

**Experimental models: Organisms/strains**		

32 male and 26 female free-ranging chimpanzees	not applicable	not applicable

**Software and algorithms**		

R 4.0.3	R Core Team, 2020	https://cran.r-project.org/
R package EloOptimized 0.3.0	Feldblum et al., 2019	https://cran.r-project.org/web/packages/EloOptimized/index.html
R package lme4 1.1-27	Bates et al., 2015	https://cran.r-project.org/web/packages/lme4/index.html
R package MuMIn 1.43.17	Bartoń, 2020	https://cran.r-project.org/web/packages/MuMIn/index.html
R package amen 1.4.4	Hoff et al., 2020	https://cran.r-project.org/web/packages/amen/index.html
R package coda 0.19-4	Plummer et al., 2006	https://cran.r-project.org/web/packages/coda/index.html
R package igraph 1.2.6	Csárdi & Nepusz, 2006	https://cran.r-project.org/web/packages/igraph/index.html
R package lavaan 0.6-8	Rosseel, 2012	https://cran.r-project.org/web/packages/lavaan/index.html
R package semPlot 1.1.2	Epskamp, 2019	https://cran.r-project.org/web/packages/semPlot/index.html

### Resource availability

#### Lead contact

Further information and requests for code and data should be directed to and will be fulfilled by the lead contact, Joseph T. Feldblum (feldblum@umich.edu).

#### Materials availability

This study did not generate new unique reagents.

#### Data and code availability

• Data are available at https://github.com/jtfeld/male_sociality_RS
• All original code is available at https://github.com/jtfeld/male_sociality_RS and is publicly available as of the date of publication (see key resources table for accession code).
• Any additional information required to reanalyze the data reported in this paper is available from the lead contact upon request.

### Experimental model and subject details

#### Subjects

Subjects were 32 male and 26 female free-ranging chimpanzees (*Pan troglodytes schweinfurthii*) in the Kasekela community in Gombe National Park, Tanzania. Males were between 11.0 and 40.8 years of age, and females were between 12.3 and 43.3 years of age (see below for inclusion criteria).

#### Ethical guidelines

Data collection was approved by Tanzania National Parks, Tanzania Wildlife Research Institute, and Tanzania Commission for Science and Technology, as well as the Duke University Institutional Animal Care and Use Committee.

### Method details

Gombe National Park is located in western Tanzania on the eastern shore of Lake Tanganyika. Since 1973, local field assistants have conducted daily full-day focal follows on individuals in the Kasekela community, one of three chimpanzee communities in the park (Goodall, 1986). The observers record the party composition, social behavior, and physical location of the focal individual and all other individuals present, and the arrival and departure time of individuals joining and leaving the focal's party. Since 1994, researchers have also collected fecal samples for genetic analysis (Constable et al., 2001). Chimpanzees have a social organization with high fission-fusion dynamics, meaning that party composition changes frequently and the full community is rarely together in a single party (Goodall, 1986). From December 1996 to November 2013, the start and end times, direction, and partner identity of all grooming bouts involving the focal individual were recorded in narrative notes. Before and after those dates, observers recorded the partner identity and direction of all grooming bouts involving the focal individual at five-minute intervals.

We used behavioral and genetic data from 1986 to 2014 to investigate the reproductive consequences of male sociality. We analyzed data by siring event, using association and grooming data in the year preceding each siring event to predict the likelihood of siring by a given male. We chose year-long intervals to facilitate comparison with earlier work on sociality and adaptive outcomes in in chimpanzees and other nonhuman primates (e.g. Bray et al., in press; Bray and Gilby, 2020; Ellis et al., 2019; McFarland et al., 2017; Silk et al., 2018, 2009, 2006a, 2006b). We included males in a given siring window if they were alive and at least 11 years old on the conception date, as the youngest known successful male sire in Gombe was 11.4 years old. The community averaged 12.3 (range 9–15) such males across periods.

We began with 60 paternity assignments which were reported in earlier publications (Constable et al., 2001; Feldblum et al., 2014; Gilby et al., 2013; Massaro et al.; Walker et al., 2017; Wroblewski et al., 2009), which include detailed descriptions of genotyping and paternity assignment methods (Massaro et al.; Walker et al., 2017; Wroblewski et al., 2009). Briefly, 8–11 microsatellite loci were genotyped for mothers, offspring, and candidate males. The eight loci that are genotyped for all individuals (Barbian et al., 2018) have a mean of 10.4 alleles per locus (range: 6–16) and a mean heterozygosity of 0.828 (range: 0.744-0.907). Fathers were first identified using the exclusion principle, and then confirmed via likelihood methods in Cervus (Kalinowski et al., 2007). All candidate males have been genotyped since 1992 for Kasekela and since 2003 for Mitumba (the northern community in Gombe). Additional genotyped males from the unhabituated southern Kalande community were included in the paternity analyses, and we conservatively assumed 90% sampling of all candidate males in likelihood analyses. In all cases, the identified father was the only male that lacked allelic mismatches with the offspring's genotype given the maternal genotype (i.e. all other males were excluded by one or more loci) and was assigned at the strict confidence level of 95%. For infants with known paternity, we estimated siring date by subtracting 226 days, the mean gestation period in Gombe, from the infant's birthdate (Boehm and Pusey, 2013).

Because mating and reproduction are less likely between closely related male-female dyads in Gombe (Feldblum et al., 2014; Walker et al., 2017), we generated pairwise relatedness values between potential sires and each infant's mother using Queller and Goodnight's R (Queller and Goodnight, 1989). Genetic relatedness data were missing for some dyads because genetic sampling began in Gombe after the death of some study subjects, so we excluded four siring events for which we were missing four or more relatedness values between mothers and potential male sires. We lacked genetic data for one additional male, so we excluded him from the seven additional siring events for which he was present. Finally, we excluded two males from siring analyses because they were unable to reproduce: Pax was excluded from all siring windows because he received severe testicular injuries before puberty and was rendered sterile, and Goblin was excluded as a potential sire from siring events after October, 1989, when he received severe testicular injuries in a fight and was subsequently suspected of being sterile (Goodall, 1986, 1992). This resulted in a dataset of 56 total siring events, with 32 unique males and 25 unique females.

Because researchers differ in their approaches to characterizing social relationships (Ellis et al., 2019; Ostner and Schülke, 2018; Silk et al., 2013; Thompson, 2019) and because different measures of social integration predict fitness-related outcomes across studies, with no obvious way to know a priori the best measure to use (Ellis et al., 2019; Ostner and Schülke, 2018), we followed recent precedent (Bray et al., in press; Ellis et al., 2019) by testing several measures of male sociality.

To measure dyadic association in each siring window we calculated dyadic Simple Ratio Index (“SRI”) values based on joint arrivals in parties of four or fewer individuals (Bray and Gilby, 2020; Cairns and Schwager, 1987; Foerster et al., 2015). The SRI accounts for the total arrivals of both members of a dyad, and therefore controls for differences in observation effort and gregariousness among individuals. We scored individuals as arriving together if they were both present at the start of a focal follow or if they joined a party with the focal individual within five minutes of each other (Bray and Gilby, 2020; Foerster et al., 2015). Males in the sample had a mean of 41.5 such arrivals per year (range: 3 to 133), with 679/740 yearly male arrival counts of more than 20 arrivals. Dyadic SRIs had a mean value of 0.03 (range 0–0.66). SRI values based on arrivals in groups of different sizes were positively correlated, and restricting joint arrivals to small groups increases the likelihood that associating individuals were actually interacting (Newman, 2001) and more accurately reflects partner choice. In addition, restricting our data to individuals' first arrival of a given day mitigates problems of autocorrelation in association data (Bray et al., in press; Bray and Gilby, 2020; Foerster et al., 2015) and the confounding effects of individual variation in gregariousness (Foerster et al., 2015; Pepper et al., 1999).

First, we used dyadic SRI values to generate four measures of individual male bondedness and social integration that have been used in previous studies of male social behavior in this population (Bray et al., in press). To measure total association, we calculated 1) the total number of association partners in each window (i.e. the total number of males with whom a given male arrived in a small group at least once), and 2) the sum of the SRI values for all association partners. To determine whether males benefitted from stronger relationships in particular, we classified strong association ties as those with an SRI value above the siring window mean (Ellis et al., 2019; McFarland et al., 2017; Silk et al., 2018), and calculated 3) the total number of strong association ties (Ellis et al., 2019; McFarland et al., 2017; Silk et al., 2018), and 4) the sum of the SRI values for strong association ties (Ellis et al., 2019) (Table 1). Because the number of males varied between periods we standardized all values by Z-transformation within each siring window.

Second, we calculated several measures of grooming effort and grooming relationship strength with other males. Males groomed other males for a mean of 506 (range 0–3990) minutes per siring window. This corresponded to males grooming a mean of 1.2% (range 0–17.7%) of their time in groups with at least one other male when one of the two males was the focal individual. Male-male dyads groomed for a mean of 46 (range 0–1105) minutes per siring window. Male-male dyads groomed at a mean rate of 0.6% (range 0–11.7%) of their focal time together. To capture total grooming effort, we included 1) a measure of total time each male spent grooming with other males, and 2) individual grooming rate, defined as the total time spent grooming other males divided by the total focal minutes with at least one other male, and 3) total number of grooming partners. We next calculated total time each dyad spent grooming and used these values to generate two individual sociality indices: 4) the number of strong grooming ties (i.e. those with grooming durations above the siring period mean) (Ellis et al., 2019; McFarland et al., 2017; Silk et al., 2018), and 5) the sum of grooming durations with partners with above-average grooming durations (Ellis et al., 2019). Finally, we calculated dyadic grooming rates by dividing dyadic time spent grooming by the total time both individuals were in a group together and one was focal (because grooming was only recorded for focal individuals). Thus this measure controls for *opportunity* to groom, ensuring that results were not driven by spatiotemporal clustering within the community range. Using these dyadic rates, we determined three additional measures of grooming: 6) mean grooming rate across all group members, 7) number of partners with a grooming rate above the siring period mean (Ellis et al., 2019; McFarland et al., 2017; Silk et al., 2018), and 8) sum of the grooming rate values for partners with above-average grooming rates (Ellis et al., 2019) (Table 1). Because of differing number of males between periods, and because the method of recording grooming changed during the study period (see above), we standardized all grooming measures by Z-transformation within each period. Dyadic SRI values were weakly positively correlated with grooming times (R^2^ = 0.09) and grooming rates (R^2^ = 0.04), indicating that the association and grooming measures captured different aspects of dyadic relationships.

Third, following studies of sociality and fitness in other taxa (Schülke et al., 2010; Silk et al., 2009), we generated several measures of social bonds using a composite index of grooming and association in small groups. To generate dyadic composite sociality indices (“CSIs”), we first scaled SRI and grooming rate values by dividing them by their respective means in each window, then averaged the scaled SRI and grooming values to generate a composite index. From these CSI values, we generated three measures of individual sociality: 1) several studies in cercopithecine monkeys have found that the strength of individuals' top three CSI scores predicted survival and reproductive output (Ostner and Schülke, 2018; Schülke et al., 2010; Silk et al., 2009), so following these we calculated the sum of each individual's top three CSI values in each period, 2) following studies of the adaptive benefits of strong and weak ties in cercopithecine monkeys (Ellis et al., 2019; McFarland et al., 2017; Silk et al., 2018), we calculated the count of strong CSI values, i.e. those above the siring window mean, for each individual, and 3) the sum of each individual's strong CSI values (Table 1). Because the number of males varied between periods we standardized all values by Z-transformation within each siring window. Details of bivariate correlations between sociality predictors are presented in Figure S6.

To ensure that poor sampling did not influence rate-based measures of sociality, we excluded dyads observed for less than 1800 min together in a given window, or for whom one individual had 20 or fewer arrivals in a window, from individual-based measures of sociality (Bray et al., in press; Bray and Gilby, 2020; Foerster et al., 2015). We also excluded dyads observed together for less than 600 min when one or the other was the focal individual from the calculation of grooming rate and CSI-based measures above.

We extracted instances of coalitionary aggression from detailed narrative field notes. Aggression events were instances of directed displays, chases, or contact aggression, and were considered coalitionary if two or three males directed aggression at one or more males (Gilby et al., 2013). Coalitions of three males were scored as three dyadic coalitions.

We generated dominance rank scores from records of submissive pant-grunt vocalizations, a formal vocal signal of submission in chimpanzees (Bygott, 1979; Goodall, 1986). To measure dominance rank, we calculated male rank scores on the date of each siring event using a modified Elo score method, with the *k* parameter and entry scores optimized using maximum likelihood fitting using the “EloOptimized” package (Feldblum et al., 2019; Foerster et al., 2016). All analyses were conducted in R version 4.0.3 (R Core Team, 2020). This method dynamically updates rank scores at each interaction, allowing for the calculation of rank on a given date. Elo scores were based on 7050 pant-grunts with unambiguous actor and recipient among 37 males age 10 or older from 1978 to 2014. Males in the current sample participated in a mean of 506 pant-grunt events (range 66–2430), which meets the robustness criterion for estimating reliable dominance hierarchies (Sánchez-Tójar et al., 2018). Because the mean value of Elo scores varied slightly between periods due to males entering the hierarchy and dying, we standardized Elo scores within each period by Z-transformation.

Finally, to make effect sizes easier to compare, we centered and scaled male age by Z-transformation.

### Quantification and statistical analysis

#### Sociality and siring success

For our model comparison procedure, we first chose a null model using an information-theoretic model selection procedure. We specified a generalized linear mixed model (“GLMM”) with a logit link function, with a binomial outcome variable indicating male siring success in each siring window (1 for the successful male, 0 for the other males) and five predictor terms: male relatedness with each mother, scaled male Elo score, scaled male age, scaled male age squared, and the count of males of reproductive age on the date of siring, as well as random intercepts for male identities. We ran this full model in the lme4 package in R (Bates et al., 2015; R Core Team, 2020), and then used a model selection procedure with corrected AIC values (“AICc”) as our selection criterion to determine the best set of non-social terms for predicting male siring success using the MuMIn package in R (Bartoń, 2020; Burnham and Anderson, 1998). AIC values can select overly complex models (Burnham and Anderson, 1998); indeed adding a nuisance (i.e. completely random) parameter to a well-fitting model will tend to produce a ΔAIC value of ∼2, potentially leading to the inclusion of the model in the top model set (Arnold, 2010). Therefore excluding models from the top model set that differ from better-supported models by the addition of one term can improve inference and parameter accuracy (Harrison et al., 2018; Richards et al., 2011). We therefore excluded such models from the set of candidate models and included models within 6 AICc points of the best model in the top model set. The best-fit model included three terms: genetic relatedness, Elo score, and male age, and accounted for 48% of the Akaike weight. The next best model (ΔAICc = 0.67) included two terms: genetic relatedness and Elo score, and accounted for 34% of the Akaike weight. We treated the best-fit model (including scaled male age, genetic relatedness with the mother, male Elo score, and random intercepts for male identity) as the non-social Null model against which we compared subsequent models.

Next, we tested the hypothesis that male sociality facilitates siring success by comparing the Null model to the set of 13 models that included all terms from the Null model, plus one measure each of male social connectedness (see above), using AICc as our selection criterion (Table 1). This procedure asked whether including a given measure of male sociality increased the likelihood of minimizing information loss when predicting siring success relative to models including no social connectedness predictor. We also calculated Akaike weights for each model, which can be interpreted as conditional probabilities for each model (Burnham and Anderson, 1998).

We found that two models including measures of association had lower AICc values than the Null model, and together accounted for 88% of model weights (see Results and Table 1). These included the count and total strength of strong association ties, respectively (see Results and Table 1). We conducted an additional set of analyses to test whether the relationship between strong association ties and siring success was driven by male gregariousness. To do this, we calculated a measure of gregariousness for each male by summing the dyadic time each male was observed with other males and females of reproductive age (males older than age 10, females older than age 11; Walker et al., 2018), and then standardized these values within each siring window by Z-transformation. We then added this term to the Null model and re-ran our model comparison procedure described above. We again compared models using corrected AIC and Akaike weights.

Our measure of dyadic association controlled for differences in observed arrivals between males (Cairns and Schwager, 1987). To confirm that our choice of index did not bias our results, we regenerated our association measures using raw joint arrival counts, rather than the Simple Ratio Index. We then re-calculated the two measures of individual association found to be important for predicting siring success in our primary analysis, this time using these raw joint arrival counts; 1) the total number of strong association ties (i.e. ties in which dyads had more joint arrivals than the yearly mean) (Ellis et al., 2019; McFarland et al., 2017; Silk et al., 2018), and 2) the sum of the joint arrivals for strong association ties (Ellis et al., 2019) (Table 1). Because the number of males varied between periods we standardized all values by Z-transformation within each siring window.

We then conducted a reduced version of the model comparison procedure from the primary analysis (see above). We compared the null model (which included scaled male age, genetic relatedness with the mother, male Elo score, and random intercepts for male identity) with two models including all terms from the null model, plus measures 1) and 2) above. Results were similar to those presented in the primary analysis, with the model including number of strong ties based on raw joint arrival counts fitting better than the null model (although the sum of joint arrivals across strong association ties did not fit better than the null model; Supplemental information and Table S1).

#### Testing the alpha concessions hypothesis among subordinate males

Finding no confounding effect of gregariousness, and consistent results whether using an association index or raw data, we next conducted a series of analyses to investigate the mechanisms by which male sociality might influence their reproductive success. First, we conducted a model comparison procedure to determine whether subordinate male affiliative relationships with the alpha male in each siring window predicted siring success. To do this, we excluded the alpha male in each window from the dataset, and further excluded 11 siring events when the alpha male was the sire. The resulting dataset included 45 siring events, with 23 unique males and 23 unique females. We again specified a binomial GLMM, with rank, age, and genetic relatedness terms as predictor variables, random intercepts for male identity, and siring success as the binary outcome variable, as our Null model. We tested the hypothesis that strong bonds with the alpha male facilitate reproductive concessions by comparing the Null model to models including all terms from the Null model plus 1) measures of bond strength with the alpha, 2) the term from our best model for predicting siring success (count of strong association ties), and 3) models including both (Table 2). As before we compared models using corrected AIC and Akaike weights. To confirm that our models met model assumptions of predictor term independence, we calculated variance inflation factors (VIFs) for each of the models in the model comparison set shown in Table 2. No term in any of the models had a VIF value above 2, indicating satisfactory predictor term independence.

We conducted two additional analyses to further assess the independence of the two mechanisms for achieving siring success suggested by the primary analyses. First, we recalculated the count of strong association ties measure, this time excluding alpha males from the count of strong association ties. This measure thus represented the number of strong ties formed by subordinate males with other subordinate males. We then re-ran the above model comparison procedure to determine whether strong bonds with the alpha male, as well as the number of strong affiliation ties with males other than the alpha*,* facilitated higher reproductive output among subordinate males. To do this we compared the Null model to models including all terms from the Null model plus 1) measures of bond strength with the alpha, 2) the count of strong association ties (excluding the alpha male), and 3) models including both (Table 2). As before we compared models using corrected AIC and Akaike weights. The new measure of the count of strong association ties was not correlated with the strength of the CSI with the alpha male (Pearson correlation = −0.0001). Model comparisons revealed the same relative model fits as reported in the primary analysis (Supplemental information and Table S2).

Second, to determine whether bonds with alpha males are functionally different from other close bonds, we ran an additional analysis to assess whether social bonds with the second-ranked male were also associated with increased siring success. If bonds with the alpha male do not differ functionally from those with other males, and bonds with the alpha were incidental to a male strategy of forming many strong bonds, we predicted that males with strong bonds with the beta male should also be more likely to sire offspring. To test this, we calculated each male's 1) association rate, 2) grooming rate, and 3) Composite Sociality Index with the beta male in each reproductive window as described above. As in the primary analysis, we excluded four siring events for which we were missing four or more relatedness values between mothers and potential male sires. We lacked genetic data for one additional male, so we excluded him from the seven additional siring events for which he was present. Finally, we excluded Pax from all siring events and Goblin from siring events after October, 1989, because they were unable to reproduce (see above for more details). We then excluded all beta males from the analysis, as we could not quantify their relationship to themselves, and excluded three siring events where the sire was the beta male. This resulted in a dataset of 53 total siring events, with 24 unique males and 24 unique females.

We then re-ran our Null model using the new dataset. As in the primary analysis, this was a GLMM with a logit link function, with a binomial outcome variable indicating male siring success in each siring window. The Null model included scaled male age, genetic relatedness with the mother, and male Elo score as predictor terms, as well as random intercepts for male identity. We then proceeded with a reduced model comparison procedure to determine whether adding each of our three measures of bond strength with the beta male improved model fit (Supplemental information and Table S3).

Finally, to better understand the complex interactions between predictor terms and siring success among subordinate males, we fit a path model using the dataset of subordinate males. As before, this dataset included 45 siring events, with 23 unique subordinate males and 23 unique females. We conducted the analysis using the lavaan package in R (Rosseel, 2012), using the default diagonally-weighted least squares estimator. We specified male Elo score, male age, and male genetic relatedness with the female as predictor (i.e. exogenous) variables, with count of strong association ties as an intermediate outcome (i.e. endogenous) variable predicted by Elo score and age, CSI with the alpha male as a second intermediate (i.e. endogenous) variable predicted by Elo score, age, and count of strong association ties, and siring success as a binary outcome (i.e. endogenous) variable predicted by all five other terms (Figure S7). The chi-square test was non-significant and root-mean-square error of approximation (RMSEA) was <0.05, indicating acceptable fit (Hu and Bentler, 1999; Steiger, 2007). This was supported by additional tests of model fit (Comparative Fit Index = 1.00, Tucker-Lewis Index = 1.057). We then used the semPlot R package (Epskamp, 2019) to visualize the associated path diagram.

#### Testing the coalitionary support hypothesis

We conducted two analyses to investigate the relationship between association in small groups and coalition formation, using a dataset of 265 male-male coalitions targeting other males from 1994 to 2012. The dataset contained a yearly mean of 14 coalitionary events (range 4–34). Following previous analyses of coalitionary behavior in the Gombe population, we limited our coalition event data to 1) directed displays, 2) chases, and 3) physical attacks. We then included coalitionary aggression events wherein two or three males simultaneously directed aggression at between one and three unambiguous male targets (Gilby et al., 2013). First, we used an Additive and Multiplicative Effects model to examine the relationship between affiliative behavior and coalition formation in one-year windows from 1994 to 2012 (Hoff, 2009; Hoff et al., 2013). For each dyad in each year, we used a binary outcome variable indicating whether the dyad formed at least one coalition during the year. We then calculated time observed in the same party, joint arrivals in parties of four or fewer individuals, and grooming rate as detailed above. Because dyadic affiliation and coalition formation may be influenced by dyadic age and rank similarity (e.g. Mitani et al., 2002), we also included terms for dyadic difference in cardinal Elo scores and dyadic age difference. We standardized all dyadic measures by Z-transformation within each year. Finally, we included individual predictor terms for age category (young: age <20 years old; prime: 20 ≤ age <30; old: age >30) and individual cardinal Elo score. The Additive and Multiplicative model approach uses a Bayesian modeling framework to analyze dyadic network data, and solves the problem of specifying random effects structures for non-directional dyadic data by representing the data as a relational matrix and estimating individual row and column effects, which are constrained to be identical for symmetric outcome variables. Row and column effects in this case captured individual heterogeneity in tendency to form coalitions. We conducted modeling in the “amen” package, which uses Bayesian model fitting algorithms to estimate AME models (version 1.4.4; Hoff et al., 2020) in R version 4.0.3 (R Core Team, 2020) using a binary probit AME model with the model specification that accounts for repeated observations, MCMC chain length of 90,000 iterations, and a burn-in period of 1000 iterations. To ensure that the MCMC chains mixed properly, we ran each model three times with different random seeds in each case, and then calculated scale reduction factors (Brooks and Gelman, 1998; Gelman and Rubin, 1992) using the R package coda (version 0.19-4; Plummer et al., 2006). The potential scale reduction factors for all parameters were ≤1.01 (all upper C.I. values ≤1.03), and multivariate potential scale reduction factors were ≤1.03, indicating sufficient convergence. Estimates and standard deviations across repeated model runs were nearly identical, but because they varied slightly, results reported in the manuscript represent those values averaged across the three repeated model runs.

Next we tested the relationship between betweenness in the network of coalition formation and count of strong association ties. To prevent pseudoreplication of observations due to overlapping siring windows, for each individual we re-calculated count of strong association ties and betweenness in the coalitions network in one-year windows between 1994 and 2012. Following Gilby et al. (2013) we used coalitions of two and of three males to generate our networks, and we treated coalitions of three males as three pairwise coalitions. While this approach likely influences triadic closure in the ensuing coalitions networks, and thus may reduce inter-individual differences in betweenness centrality, our goal in this analysis was to determine whether male bonds might be responsible for the previously-reported relationship between coalitionary betweenness and siring success (Gilby et al., 2013), and thus we kept methods consistent. We then excluded years from the analysis in which fewer than 8 dyads formed coalitions with each other to ensure sufficient network density to calculate differentiated betweenness values. This resulted in a dataset with 13 years of data, with 154 observations of 19 unique males. We calculated betweenness in the network of coalition formation in each window using the igraph package in R (Csárdi and Nepusz, 2006; R Core Team, 2020). We scaled counts of strong association ties within each year via Z-transformation to account for varying community size between years, and then ran a simple linear model to examine the relationship between each individual's count of strong association ties and their coalitionary betweenness.

#### Supervising bodies

We thank Jane Goodall for permission to work with the long-term data and the Tanzania National Parks, Tanzania Wildlife Research Institute, and Tanzania Commission for Science and Technology for permission to work in Gombe National Park. All data collection was approved by these Tanzanian bodies and the Duke University Institutional Animal Care and Use Committee.

## Footnotes

Supplemental information can be found online at https://doi.org/10.1016/j.isci.2021.102864.
