# Supplementary material for: Social bonds provide multiple pathways to reproductive success in wild male chimpanzees
Source: iScience. 2021 Aug 17;24(8):102864. doi: 10.1016/j.isci.2021.102864 (PMC8390850; doi:10.1016/j.isci.2021.102864)
Supplement: Document S1. Supplemental methods, Figures S1–S7, Tables S1–S4 [file mmc1.pdf]

iScience, Volume *24*

Supplemental information

Social bonds provide multiple pathways to reproductive success in wild male chimpanzees

Joseph T. Feldblum, Christopher Krupenye, Joel Bray, Anne E. Pusey, and Ian C. Gilby

Supplementary material

Results of supplementary analysis

Supplementary analysis 1: using raw counts of joint arrivals

In this analysis, we replaced the Simple Ratio Index of joint arrivals in small groups with raw joint arrival counts (see STAR Methods for details). Results were similar to those presented in the primary analysis, with the model including number of strong ties based on raw joint arrival counts fitting better than the null model (although the sum of joint arrivals across strong association ties did not fit better than the null model; Table S1). As in the primary analysis, according to the best model, males with more strong association ties had a higher likelihood of siring offspring (binomial GLMM, odds ratio = 1.38, 95% confidence interval = [1.01 – 1.89]); a one standard deviation increase in the number of strong association ties corresponded to a 38% increase in probability of siring a given offspring.

Supplementary analysis 2: excluding alpha males from count of strong association ties

In this analysis, we excluded alpha males from counts of strong association ties among subordinate males (see STAR Methods for details). The new measure of the count of strong association ties was not correlated with the strength of the CSI with the alpha male (Pearson correlation = -0.0001). Model comparisons revealed the same relative model fits as reported in the primary analysis; the best-fit model (ΔAICc = -12.05) included the composite sociality index (CSI) of grooming and association with the alpha male as well as the count of strong association ties, while the next best fitting model included grooming rate with the alpha male as well as the count of strong association ties (ΔAICc = -9.95; Table S2). In the best-fit model, both CSI with the alpha male (OR = 1.50, CI = 1.07 – 2.10) and count of strong association ties (OR = 1.79, CI = 1.26 – 2.53) were positively associated with likelihood of siring offspring. The model also accounted for male age, male Elo score, and male genetic relatedness with the mother.

Supplementary analysis 3: Do bonds with the beta male have similar effects as those with alpha males?

In this analysis, we generated measures of association, grooming, and a Composite Sociality Index for each male with the beta male rather than the alpha male (see STAR Methods for details). We were unable to directly compare model fits with those in the “bonds with alpha males” analysis because the data sets differed. However, in the primary analysis, models including all measures of bond strength with the alpha male fit better than the Null model (see Table 2). On the other hand, all models including terms for bond strength with the beta male fit worse than the Null model (Table S3). Further, none of the measures of bond strength with the beta male in the model set were positively associated with likelihood of siring offspring (association: Odds Ratio = 0.91, 95% confidence interval = [0.66 – 1.24]; grooming: OR = 0.92, CI = [0.67 – 1.27]; CSI: OR = 0.96, CI = [0.70 – 1.31]).

Males that formed strong bonds with the alpha male were more likely to sire offspring, whereas those that did so with the beta male were not. This supports the idea that bonds with the alpha represent a different route to siring success than forming many strong association ties.

Supplementary tables

Affiliative index	Model	df	AICc	ΔAICc	Akaike weight
Joint arrivals	Count of strong association ties	6	365.374	-2.079	0.610
(none)	**Null model**	5	367.453	0	0.216
Joint arrivals	Sum of joint arrivals among strong assn. ties	6	367.884	0.430	0.174

Table S1. Model comparisons using joint arrival counts rather than the Simple Ratio Index, related to Results, Supplementary results, and Table 1: List of models and model fit parameters for full data set, using raw joint arrival counts instead of SRI based on joint arrivals. Models include all terms from the null model, plus the term described in the Model column. ΔAICc shows difference in corrected AIC score between each model and the null model, with negative values indicating models that fit better than the null model and positive values indicating those that fit less well.

Model	df	AICc	ΔAICc	Akaike weight
CSI with alpha + count of strong association ties (no alpha)	7	282.141	-12.048	0.593
Grooming rate with alpha + count of strong association ties (no alpha)	7	284.237	-9.952	0.208
Count of strong association ties (no alpha)	6	285.492	-8.697	0.111
Association rate with alpha + count of strong association ties (no alpha)	7	286.26	-7.929	0.076
CSI with alpha	6	291.437	-2.752	0.006
Association rate with alpha	6	292.645	-1.544	0.003
Grooming rate with alpha	6	292.848	-1.341	0.003
**Null model**	5	294.189	0	0.001

Table S2. Model comparisons after excluding alpha males from counts of strong association ties, related to Results, Supplementary results, and Table 2: List of models and model fit parameters using measures of social relationships to predict male siring success among subordinate males only. In this analysis, counts of strong association ties counts ties with other subordinate males *only*. Models include all terms from the null model, plus the term(s) described in the Model column. ΔAICc shows difference in corrected AIC score between each model and the null model, with negative values indicating models that fit better than the null model.

Model	df	AICc	ΔAICc	Akaike weight
**Null model**	5	334.057	0	0.452
Association rate with beta	6	335.735	1.678	0.195
Grooming rate with beta	6	335.853	1.796	0.184
CSI with beta	6	336.036	1.979	0.168

Table S3, Comparisons of models including measures of bond strength with the Beta male, related to Results, Supplementary results, and Table 2: List of models and model fit parameters using measures of social relationships with the beta male to predict male siring success among males other than the beta male. Models include all terms from the null model, plus the term described in the Model column. ΔAICc shows difference in corrected AIC score between each model and the null model, with negative values indicating models that fit better than the null model.

Term	Estimate (undstandardized)	s.e.	*p*
N strong assn. ties ~			
Male Elo	0.063	0.06	0.296
Male age	0.101	0.056	0.069
CSI with alpha ~			
Male Elo	0.382	0.059	0.000
Male age	0.089	0.046	0.055
N strong assn. ties	0.025	0.038	0.502
Sire ~			
Male age	-0.151	0.105	0.147
Male Elo	0.052	0.108	0.632
Male-female relatedness	-1.222	0.46	0.008
CSI with alpha	0.189	0.082	0.022
N strong assn. ties	0.286	0.084	0.001

Table S4, results from path analysis of sociality and siring success among subordinate males, related to Results, Figure S7. Results from path analysis of factors predicting male sociality and reproductive success among subordinate males. See STAR Methods for model details.

Supplementary figures:

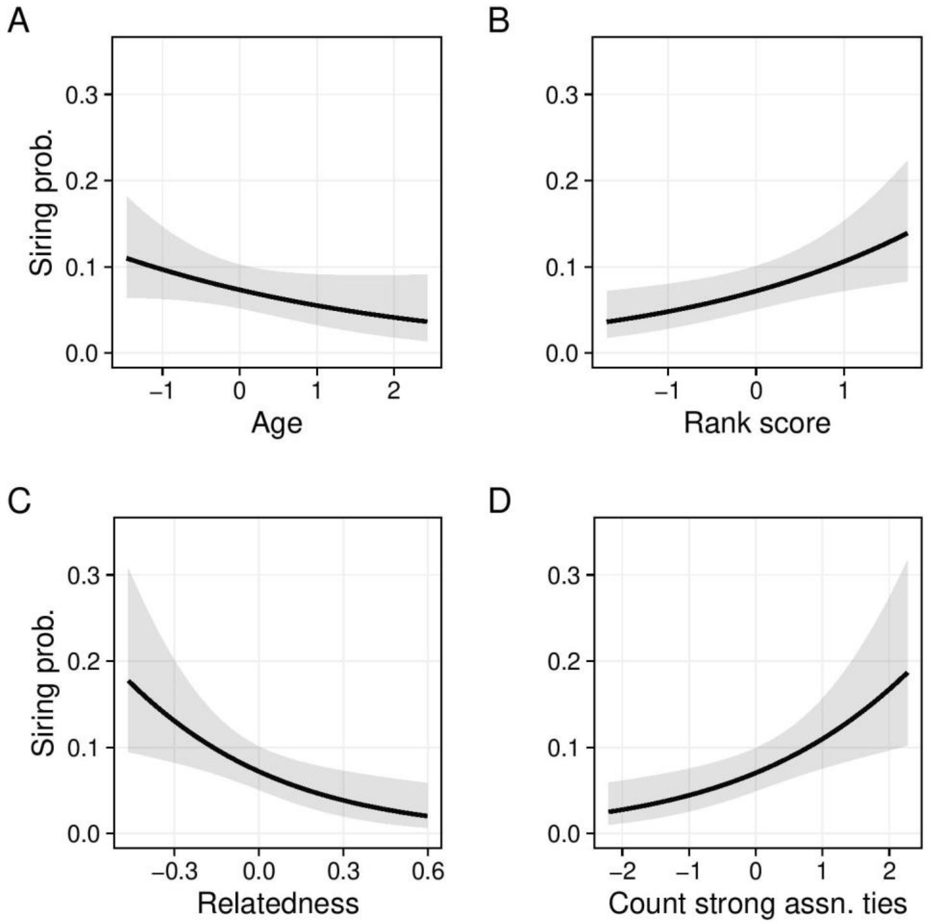

Figure S1: Predictors of siring success among all males, related to Results and Figure 1: Predicted relationship between siring probability and **A** male age; **B** male rank score; **C** male-female genetic relatedness; and **D** count of strong association ties, using the full dataset of 56 siring events. Age is standardized across the entire dataset, while Rank score and Count of strong association ties are standardized within period. Error regions indicate 95% confidence intervals.

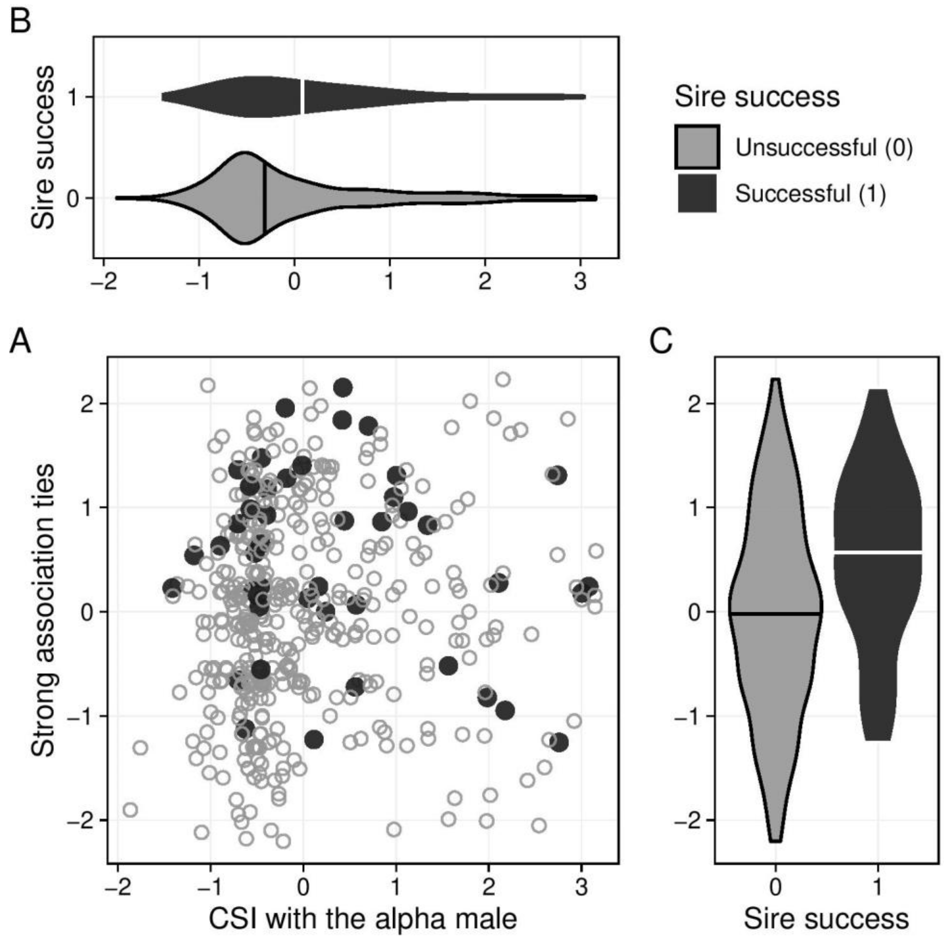

Figure S2: Strong association tie count, CSI with the alpha, and siring success, related to Results and Figure 2: Empirical distribution of scaled strong association tie count and composite sociality index (CSI) with the alpha male by siring success, among males ranked second or lower. These two measures were weakly positively correlated (Pearson correlation = 0.08). **A** shows the bivariate relationship between the two predictor terms, and **B** and **C** show the distribution of CSI with the alpha male and Strong association tie count, respectively, among males that sired and males that failed to sire offspring. Successful sires are represented with dark gray filled circles in **A** and dark grey violins in **B** and **C**, while males that failed to sire offspring in a given window are in lighter gray empty circles in **A**, and lighter gray violins in **B** and **C**. Lines through violins indicate 50% quantiles.

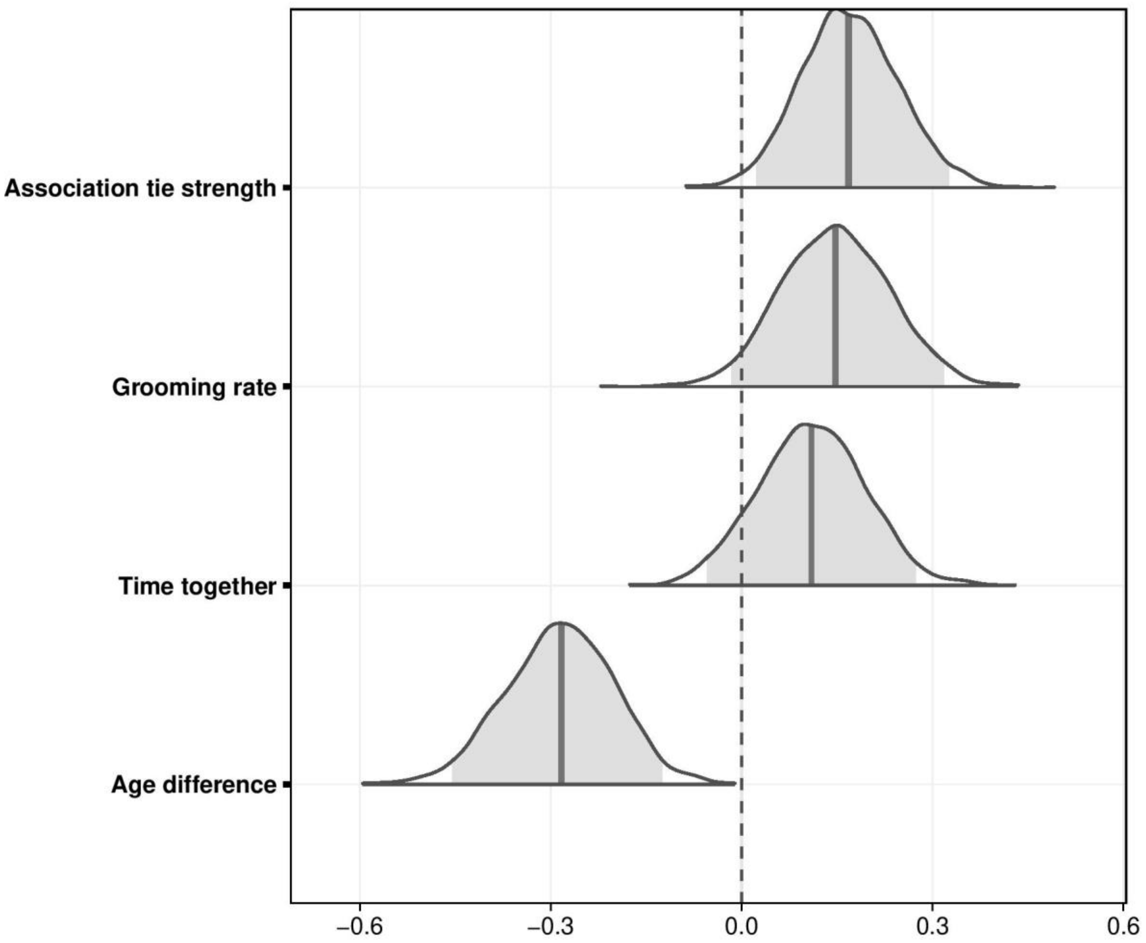

Figure S3: Posterior predictive distributions of terms predicting dyadic coalition formation, related to Results: Posterior predictive distributions of parameter estimates from probit AME model predicting dyadic coalition formation. Vertical lines and grey regions in each distribution show medians and 95% credible intervals, respectively. Higher association tie strength (i.e. dyads that associated more in small groups) was associated with a higher likelihood of forming at least one coalition in a given year, after accounting for dyadic grooming rates, time spent together, dyadic age difference, and other age and dominance rank factors not presented here. The negative effect of age difference indicates that males that were closer in age were more likely to form coalitions. See Methods for model description and list of all model parameters.

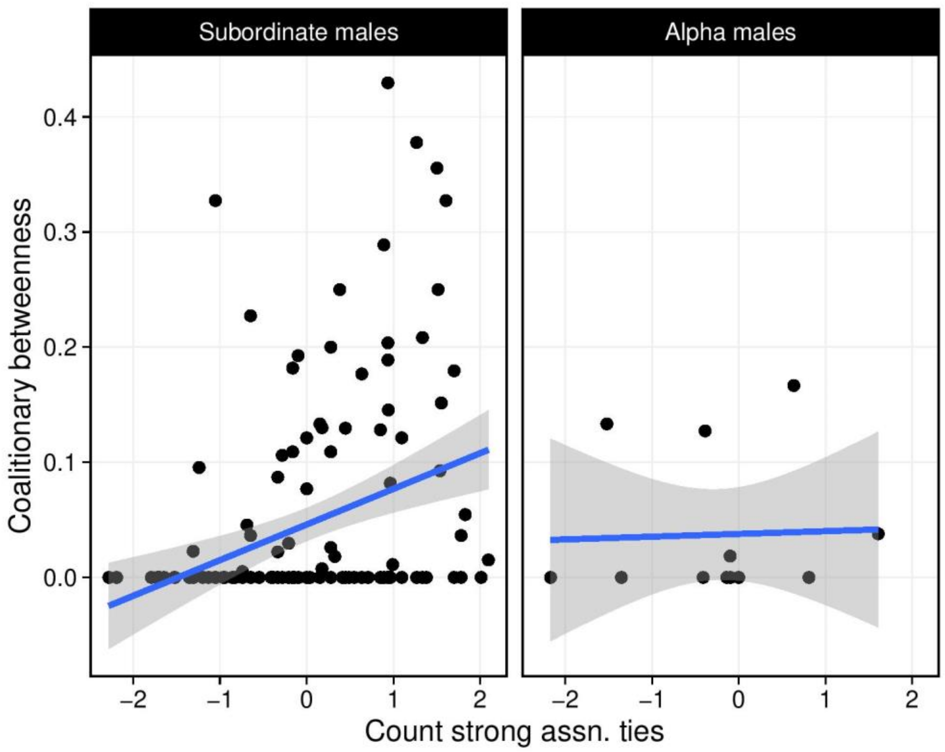

Figure S4: Strong association ties and coalitionary betweenness, related to Results: The relationship between strong association tie count and betweenness in yearly the network of coalition formation. The left-hand panel shows males ranked second and lower, while the right hand panel shows alpha males only. We excluded years with insufficient observed coalition events (see Methods). Subordinate males with more strong association ties also had higher betweenness in the network of male coalition formation. Blue lines and error regions indicate linear smooths and standard errors, respectively.

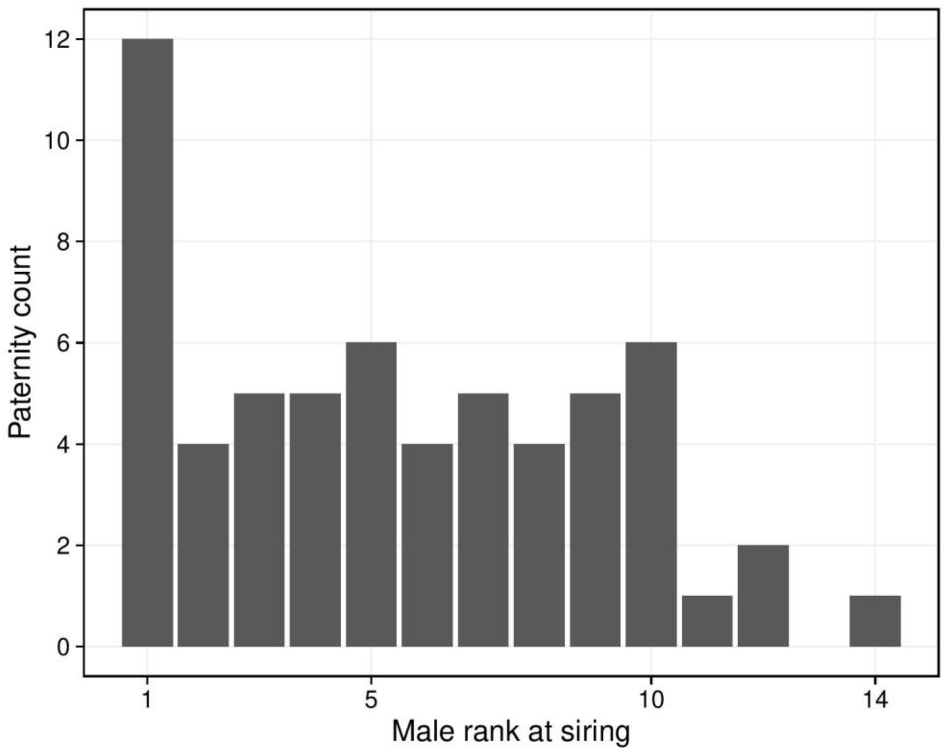

Figure S5, Distribution of siring success by ordinal rank, related to Results. Note that although alpha males (the left-hand most bar) sired a higher proportion of offspring than other males, males of ranks 2 through 10 were about equivalently successful, siring between four and six offspring.

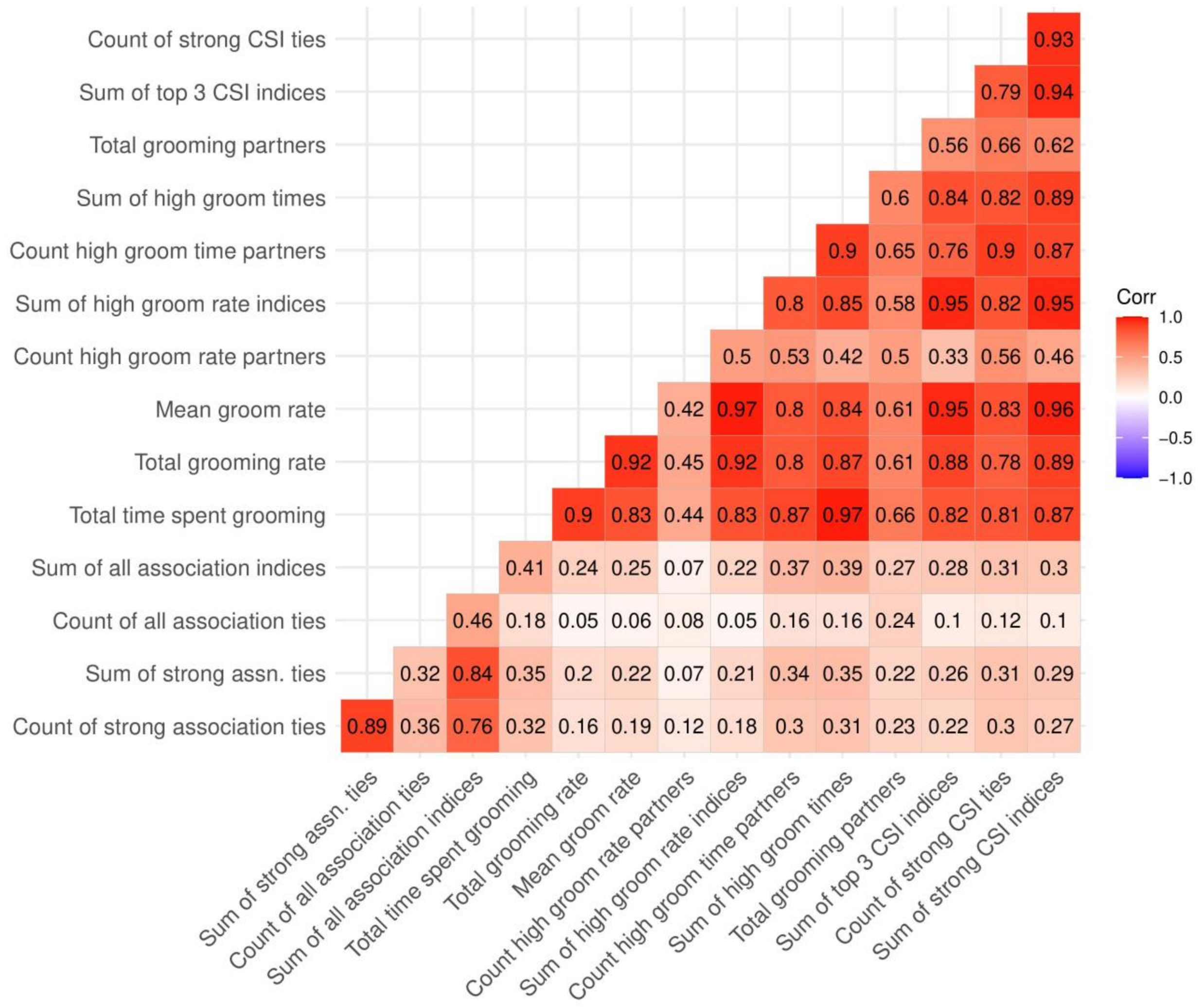

Figure S6, correlations of predictor terms, related to Methods. Pearson correlations between all sociality terms from the first model comparison analysis.

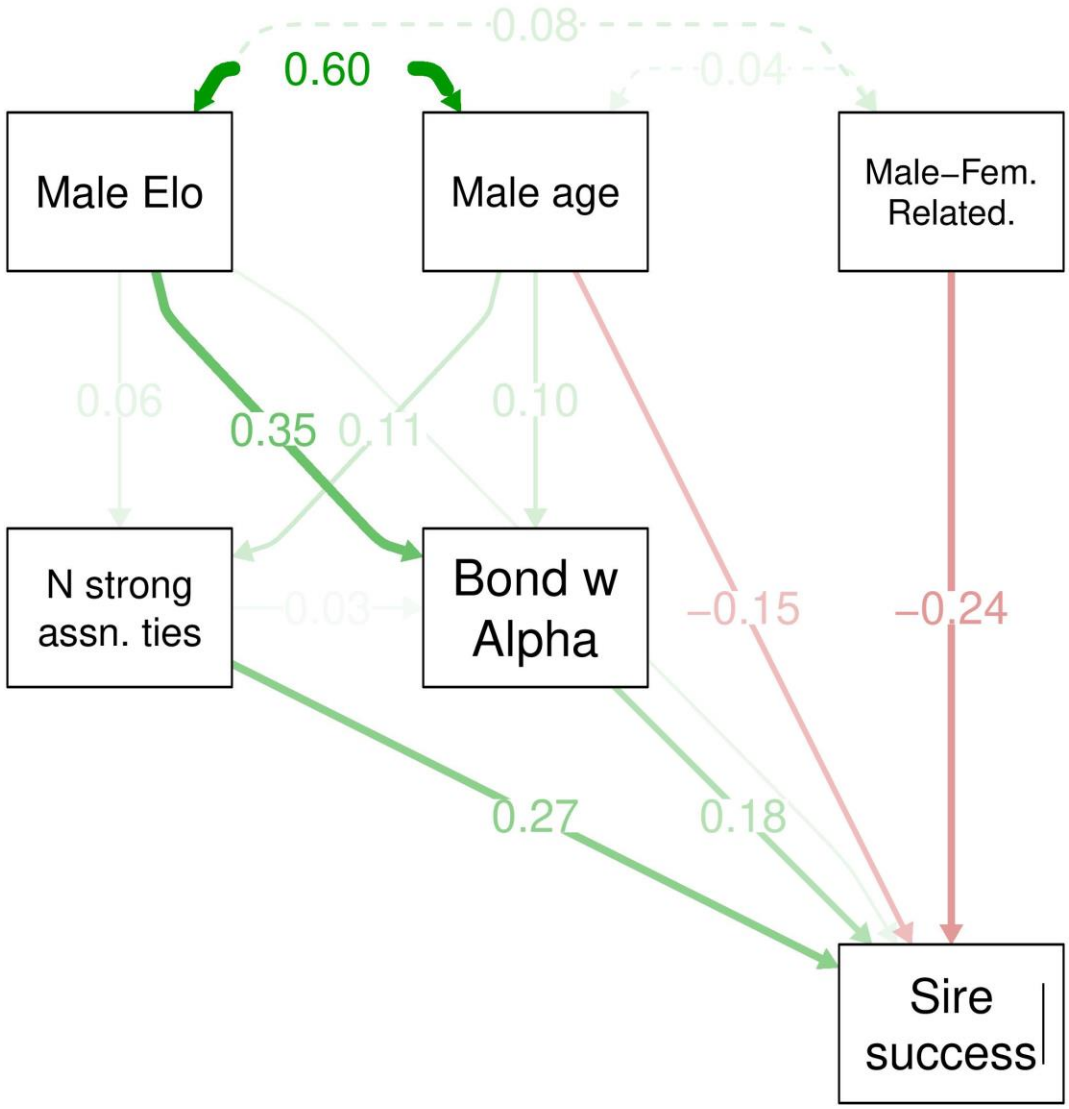

Figure S7, path diagram showing relationships between terms predicting siring success among subordinate males, related to Results. Edges with one arrow represent standardized path coefficients. Edges with bidirectional arrows represent covariances. Edge width and opacity are scaled to standardized estimates. *Male Elo* and *Male age* represent male Elo score and male age on the estimated siring date, *Male-Fem. Related.* indicates the relatedness value of each male with the offspring's mother, *N strong assn. ties* indicates the standardized count of strong association ties, *Bond w Alpha* indicates the CSI value of each subordinate male with the alpha male, and *Sire success* indicates the binary outcome variable indicating siring success. See methods for more details. The path diagram demonstrates the independent associations of bonds with the alpha male and count of strong association ties on siring success, as well as the indirect association between subordinate male Elo score and siring success, mediated by bond strength with the alpha male. It is important to note that causal relationships implied here are based on assumptions made by the authors, and that it is not possible to definitively establish causal relationships with the available data.
